# Effect of Oral Administration with *Lactobacillus plantarum* CAM6 Strain on Sows during Gestation-Lactation and the Derived Impact on Their Progeny Performance

**DOI:** 10.1155/2021/6615960

**Published:** 2021-01-08

**Authors:** César Betancur, Yordan Martínez, Guillermo Tellez-Isaias, Rogel Castillo, Xinghua Ding

**Affiliations:** ^1^Departamento de Ciencias Pecuarias, Facultad de Medicina Veterinaria y Zootecnia, Universidad de Córdoba, Montería 230002, Colombia; ^2^Departamento de Ciencia y Producción Agropecuaria, Escuela Agrícola Panamericana Zamorano, Valle de Yeguare, San Antonio de Oriente, Francisco Morazán 96, Honduras; ^3^Department of Poultry Science, University of Arkansas, Fayetteville, AR 72701, USA; ^4^School of Biological Sciences, The University of Hong Kong, Pokfulam Road, Hong Kong SAR, China

## Abstract

**Background:**

To evaluate the biological response of the sows and their offspring with oral administration of *Lactobacillus plantarum* CAM6 in breeding sows, a total of 20 Pietrain breeding sows with three farrowings and their descendants were used, randomly divided into two groups of 10 sows each. Treatments included a basal diet (T0) and basal diet +10 mL biological agent containing 10^9^ CFU/mL *L. plantarum* CAM6 (T1). No antibiotics were used throughout the entire experimental process of this study.

**Results:**

The *L. Plantarum* CAM6 supplementation in sows' feeding did not affect (*P* > 0.05) the reproductive performance of the sows; however, the number of deaths for their offspring before weaning (*P* ≤ 0.05) decreased. In addition, the oral administration of *Lactobacillus plantarum* CAM6 in sows increased (*P* ≤ 0.05) the content of lactose, nonfat solids, mineral salts, and the density of sows' milk, with a decrease in milk fat. Moreover, the probiotic feed orally to the sows improved the body weight (*P* ≤ 0.05) and reduced the diarrhea incidence of their offspring (*P* ≤ 0.05). Also, the probiotic administration of sows changed (*P* ≤ 0.05) the serum concentration of Na^+^, pCO_2_, and D-*β*-hydroxybutyrate and increased (*P* ≤ 0.05) the leukocytes, lymphocytes, and platelets in their piglets.

**Conclusion:**

Oral administration of *Lactobacillus plantarum* CAM6 in breeding sows improved body weight, physiological status, and the health of their offspring. And preparing the neonatal piglets physiologically is of great importance to the pig farming industry which could decrease the operational cost and medication (especially antibiotics) consumption of the pig producers.

## 1. Introduction

In the first few days after birth, the piglets are less stable and more susceptible to disturbances and microbial pathogens, because newborn pigs are immunodeficient at birth, and there is no transfer of antibodies through the uterus [1]. They have to acquire passive immunity from colostrum and breast milk for their survival [2]. Therefore, to minimize the use of antibiotics in piglets, one of the strategies is to prepare the offspring physiologically and microbiologically by administering functional feed orally during the perinatal period of the sows [3].

Among the verities of functional feeds taken orally, probiotics are one of them which are live microbial feed supplements with the capability to beneficially affect the host animal by improving its intestinal microbial balance [4]. Probiotics have been explored as feed additives in swine production by considering their beneficial effects on intestinal microbial homeostasis of the sows and indirectly benefiting the progeny and helping to minimize the use of subtherapeutic antibiotics [5, 6].

There are several studies reported that the performance of nutrient absorption of the sows during gestation and lactation influences the litter size and body weight of suckling pigs at birth and livability at weaning [7]. And the better utilization of nutrients during lactation is reflected in high-quality milk, which would indirectly improve the growth performance of their piglets [8].

Lactic acid bacteria (LAB) have been the main microorganisms that have been used as potential probiotics in swine production for their beneficial role in the gastrointestinal tract [9]. Few studies have been elucidated on the effect of the supplementation of *Lactobacillus* spp. in sow nutrition can extend to their offspring. Previous studies showed that the isolated *Lactobacillus plantarum* CAM6 can ferment a wide spectrum of plant carbohydrates and tolerant to bile salts and low pH; and it is antagonistic against the common Enterobacteriaceae in swine production [10, 11]. Therefore, the objective of this study was to evaluate the efficacy of *Lactobacillus plantarum* CAM6 in sows during the gestation and lactation and its influence on their milk as well as on the blood parameters and productive performance of their descendants.

## 2. Materials and Methods

The study was carried out in accordance with the National Institutes of Health guide for the care and use of laboratory animals (NIH publications No. 8023, revised 1978), and the experiment was approved by the Animal Care and Use Committee of Córdoba University and Research (Resolution No. 1 of January 26, 2016).

### 2.1. Experimental Location

The research in the swine experimental areas of the University of Córdoba, Berástegui campus, Córdoba, Colombia, located between the coordinates 7°23′9°26′ north latitude and 74°52′76°32′ west longitude meridian of Greenwich was developed. The average annual temperature is 28°C, and the average rainfall is 1400 mm per year.

### 2.2. Probiotic, Animals, and Treatments

The *L. plantarum* CAM6 strain (access number 4MK523644.1) was isolated from Creole pigs (Zungo Pelado) from the north coast of Colombia [11]. This bacterial strain grew under conditions of pH (3.0), bile salt (0.3%), NaCl (10%), and high temperatures and showed antagonistic activity against bacterial pathogens that frequently infect piglets [10, 11].

This strain was inoculated in pine, apple, banana, and papaya peel juice. Growth kinetics was performed to determine the most appropriate fruit peel concentration and optimize the substrate to the inoculum ratio and pH. The best medium consisted of 40% fruit peels and 60% water. The optimal substrate to inoculum ratio was 6.81, and the best pH was 5.29. Under these conditions, a bacterial density of 10^9^ CFU/mL was obtained, and this concentration was used as a probiotic treatment.

A total of 20 apparently healthy Pietrain sows with three farrowing were used in the last third of gestation (75 days of gestation) and throughout suckling period according to a completely randomized design with two treatments and 10 replicates. Farrows were synchronized, and no anomalies were found in the first two-thirds of the gestation.

The treatments consisted of T0—basal diet (BD) feeding group—and T1—BD+10 mL biological agent containing 10^9^ CFU/mL *L. plantarum* CAM6 feeding group. No antibiotics were added in either group. It is noted important that the probiotic was not offered to suckling piglets. The diets were formulated according to the recommendations described by NCR (2012) to satisfy the nutritional requirements of the pig category. The ingredients of the diets are shown in Table 1.

### 2.3. Experimental Conditions

The pregnant sows were placed individually in metal pens of 2.10 m × 0.80 m × 1 m. Three days prior to the expected date, farrowing sows were allocated into metallic farrowing crates of 2.10 m × 1 m × 1 m, inside of an area of 1 m × 1.50 m for their offspring. Feeding for pregnant and lactating sows was restricted at a rate of 2 kg/animal/day. The feed was supplied twice a day (8:00 am and 3:00 pm) in tubular feeders of 52 cm × 16 cm, and the water was available *ad libitum* in nipple-type automatic drinkers. From 8 days old, a prestarter diet in the form of pellets at a rate of 10 grams per animal/day was offered to the piglets, which was increased consecutively until reaching 300 grams per animal/day in two frequencies (8:00 a.m. and 3:00 p.m.) Heater lamps to manage the temperature of the suckling piglets ranged from 30°C (at input) with progressive reduction to 26°C (at output) were used.

### 2.4. Productive and Reproductive Indicators of the Sow

The individual body weight of the sows was determined at 75 days of gestation (beginning of the experiment), at farrowing and at weaning (Mettler Toledo, Digital Scale, Ohio, USA). Using these data, weight loss during farrowing and weaning was calculated. Also, the days of gestation and weaning-estrus interval were determined per sow. The following data for each litter were recorded: the number of piglets born alive and dead and specific time of death during the lactation period and weight of the piglets at 1, 7, 14, 21, and 28 days old using a precision digital ±0.1 g, scale (OSBORNE®, model 37473®, Kansas, Missouri, USA).

### 2.5. Chemical Composition of the Milk

Milk was collected in the morning prior to the suction of the piglets (7:00 a.m.), within 4 days after the initiation of farrow. The milk samples were placed in sterile bottles, previously labeled and kept refrigerated (4°C). Proteins, fats, lactose, nonfat solids, total solids, mineral salts, and density were determined using an ultrasonic milk analyzer (Biolac 60, Bogotá, Colombia). The milk tests were performed at the Laboratory of the University of Córdoba.

### 2.6. Diarrhea Index of Lactating Piglets

Diarrhea incidence (DI) in suckling piglets was determined during the experimental period using the following formula: DI = number of diarrhea/(number of animals × total days) × 100 [12].

### 2.7. Basic Acid State (BAS) and D-*β*-Hydroxybutyric Acid (BHB)

For this study and hematological profile, five 28-day-old piglets were randomized for treatment, which were identified (marked on the ear). 10 mL of blood samples was collected from the jugular vein with 21G x 1.5 needles, previous disinfection of the area, and adequate restraint. To obtain blood, serum tubes with vacutainer red cap without anticoagulant (Vacutainer®; BD, Franklin Lakes, New Jersey, USA) were used, which were centrifuged at 3000 rpm (Eppendorf centrifuge AG, New York, USA) for 15 min at room temperature. Samples were placed on ice and analyzed after 2 hours of collection.

For the analysis of the BAS, 1 mL of plasma was taken and pH, pCO_2_, Na, K, Cl, HCO_3_^−^, and anion gap were determined by means of an electrolyte and blood gas analyzer (VetSat, IDEXX, USA). In the blood serum, BHB by optical reflectometry with commercial kits (kit Rambut ® D-3-Hydroxybutyrate–RB 1007, Randox Laboratories, Crumlin, County Atrium, UK) in an ultraviolet spectrophotometer (Metrolab 1600, USA) was analyzed.

### 2.8. Hematological Profile

In blood plasma, the following hematological variables were evaluated in a semiautomatic analyzer (Horiba ABX Micros ESV 60®; Paris, France): hemoglobin, hemogram, hematocrit, mean corpuscular volume (MCV), mean corpuscular hemoglobin concentration (CMCH), mean corpuscular hemoglobin (MCH), and mean platelet volume (MPV). All the samples were processed in the Veterinary Clinical Laboratory of the Faculty of Veterinary Medicine and Zootechnics, University of Córdoba.

### 2.9. Statistical Analysis

Sow and its litter were considered the experimental units. The data were expressed as the mean, SD, and EE ±. Means were compared using Student's *t*-test; mortality and the newborn index were analyzed for comparison of proportions in SPSS 21.0 (SPSS Inc., Chicago, IL, USA).

## 3. Results

### 3.1. Effect of Lactobacillus plantarum CAM6 on Reproductive Indicators of the Sows

The oral administration of *Lactobacillus plantarum* CAM6 for sows did not affect (*P* > 0.05) the body weight (BW) of the sows in the third part of the pregnancy (Table 2), at the time of parturition or at the end of lactation, and notable difference was found for sow weight loss (*P* > 0.05). In addition, these probiotic treatments for sows did not indicate significant differences (*P* > 0.05) between treatments for the days of gestation, live piglets at birth/litter, piglets dead at birth/litter, weaned piglets/litter, and weaning-estrus interval. However, the probiotic feeding for sows decreased (*P* ≤ 0.05) the number of dead piglets prior to weaning per litter (Table 2).

**Figure 1:**
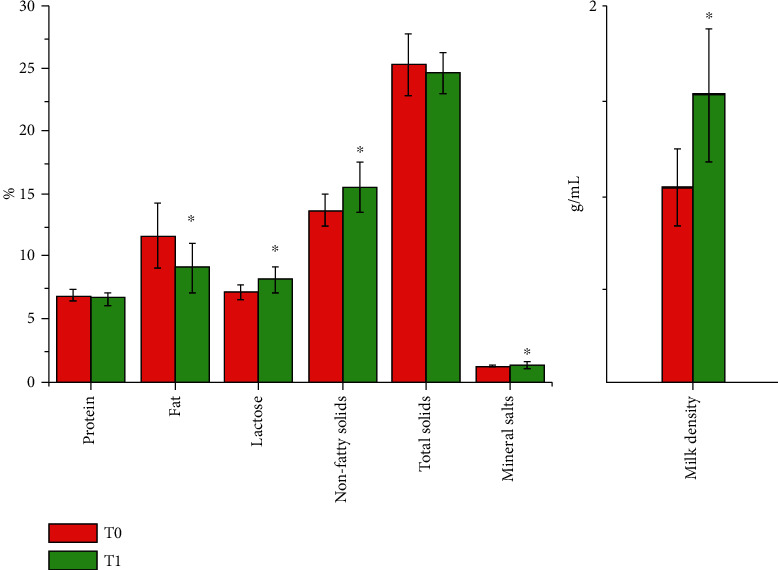
Effect of the oral administration of *Lactobacillus plantarum* CAM6 for sows on the chemical composition and density of milk produced by them. T0: basal diet (BD); T1: BD + 10 mL biological agent containing 10^9^ CFU/mL *L. plantarum* CAM6. ^∗^*P*≦0.05.

The chemical composition and density of the milk of lactating sows with oral administration treatment of *Lactobacillus plantarum* CAM6 are shown in Figure 1. The concentration of lactose, nonfat solids, mineral salts, and milk density increased (*P* ≤ 0.05) with the probiotic treatment group (T1) compared to the control group (T0). However, T1 decreased (*P* ≤ 0.05) fat by 2.53% with relation to T0. The level of protein and total solids did not differ significantly between the experimental groups (*P* > 0.05). These values were within normal limits according to Boyce et al. [13].

### 3.2. Healthy Indicators of the Piglets

**Figure 2:**
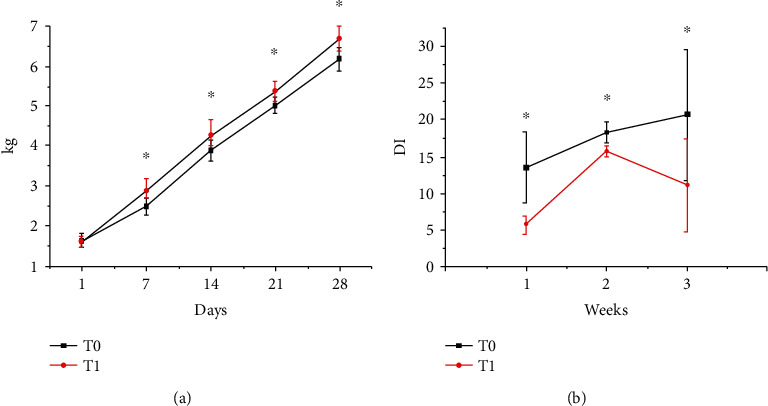
Derived impact of *Lactobacillus plantarum* CAM6 supplementation for sows to their progeny performance: (a) the derived impact of the probiotic supplementation for sows to the body weight of their progeny and (b) the derived impact of the probiotic supplementation for sows to reducing the diarrhea incidence (DI) of their progeny. T0: basal diet (BD); T1: BD + 10 mL biological agent containing 10^9^ CFU/mL *L. plantarum* CAM6. ^∗^*P*≦0.05.

The inclusion of a probiotic with *Lactobacillus plantarum* CAM6 in sows before farrowing and during lactation improved the body weight of the piglets from 7 days until weaning (28 days) with significant differences between the control (T0) and probiotic (T1) groups (*P* ≤ 0.05; Figure 2(a)). This is an important issue because the overall performance of growing-finishing pigs is determined by the performance of the first days after weaning, and this depends on the weaning weight [14]. And the oral administration with *Lactobacillus plantarum* CAM6 for sows significantly reduced the incidence of diarrhea in lactating piglets (Figure 2(b); *P* ≤ 0.05).

The *Lactobacillus plantarum* CAM6 treatment on sows did not modify (*P* > 0.05) some parameters of the basic-acid state (BAS) such as pH, K, Cl^−^, HCO_3_^−^, and anion gap in piglets. However, some parameters increased (*P* ≤ 0.05) such as Na^+^, pCO_2_, and D-*β*-hydroxybutyrate (BHB) in the T1 group (Table 3).

Supplementation of *Lactobacillus plantarum* CAM6 in sows did not change significantly (*P* > 0.05) the monocytes, granulocytes, eosinophils, erythrocytes, hemoglobin (Hb), hematocrit (Hto), mean corpuscular volume (MCV), mean corpuscular hemoglobin (MCH), and concentration of mean corpuscular hemoglobin (CMCH) in their piglets. Nevertheless, the bacterial product modified (*P* ≤ 0.05) leukocytes, lymphocytes, and platelets (Table 4). And it is also worth mentioning that all these values were within normal ranges according to Friendship et al. and Cooper et al. [15, 16].

## 4. Discussion

### 4.1. Effect of Lactobacillus plantarum CAM6 Supplementation on Reproductive Indicators of the Sows

In this study, the oral administration of *Lactobacillus plantarum* CAM6 for sows did not affect (*P* > 0.05) the body weight (BW) of the sows in the third part of the pregnancy (Table 2), at the time of parturition or at the end of lactation, and no notable difference was found for sow weight loss (*P* > 0.05). Similar results were reported by Wang et al. and Hayakawa et al. when they used different probiotics in sow nutrition [5, 17]. But, in our study, the chemical composition and density of the milk secreted by the sows were found changed. Lactation performance may be associated with lactose levels [18], because this sugar is the main source of energy in milk, which is essential for lactating piglets [19]. The higher concentration of lactose in the T1 group (*P* ≤ 0.05) compared with those in the T0 group can reflect better utilization of energy used by this group of sows. This may be due to the symbiotic relationship between *L. plantarum* and intestinal microorganisms that promotes homeostasis of the intestinal microbiome, which in turn indirectly enhances the absorption of nutrients such as polysaccharides, proteins, and lipids, thereby enhancing the gluconeogenesis in the sow [9]. Current studies suggested that gut microbiota plays a significant role in maternal metabolism [3, 20]; in this way, consumption of probiotic bacteria by lactating sows can improve the absorptive capacity of the intestinal mucosa which may increase milk concentration of certain nutrients [8], such as a higher percentage of minerals and nonfatty solids. In addition, the oral administration of *L. plantarum* for the sows decreased the fat in their lactating milk. The lower percentage of fat of milk in T1 (*P* ≤ 0.05) compared to that in the T0 group indicated the potential of *Lactobacillus plantarum* CAM6 for reducing fat in lactating milk. Certain probiotic bacteria with bile salt hydrolase (BSH) activity can lower cholesterol by enzymatically deconjugating bile acids [21]. Genes for BSH on *L. plantarum* Lp9 has been identified, which allows to reduce the absorption of cholesterol and saturated fatty acids [22].

### 4.2. Effect of Lactobacillus plantarum CAM6 Extended to the Piglets

In this study, the oral administration with *Lactobacillus plantarum* CAM6 in sows reduced their piglet mortality before weaning; perhaps the improvements in preweaning performance of the suckling piglets may have been attributed to beneficial effects of probiotics supplementation on sows' milk composition and benign microbial homeostasis in the sows' guts [19, 23]. According to Rodriguez and Chen et al., sows can exert a modulation of the intestinal microbiota of the progeny via modifying the chemical composition of the milk consumed by their descendants [24, 25]. And in our study, the beneficial effects of probiotics supplementation on sows' performance included improving the quality of milk composition. Merrifield et al. and Dou et al. reported that the establishment of a healthy intestinal microbiota in early life might be essential to promote piglets' immunity and health, with a considerable decrease in mortality [26, 27]. In addition, Martin et al. reported that orally supplying probiotics in lactating sows increased the presence of the lactic acid bacteria in the biological fluid (milk), which caused favorable changes in the gut and improved the production of antipathogenic compounds, viability, and body weight of the piglets [28]. In our study, the oral administration with *Lactobacillus plantarum* CAM6 in sows indeed significantly reduced the incidence of diarrhea of their progenies (Figure 2(b); *P* ≤ 0.05). The low incidence of diarrhea in the T1 group could possibly be due to the fact that the lactic acid bacteria produce lactic acid which are toxic to the pathogens in the gut of pigs [29]. Perhaps, our probiotic was also incorporated into breast milk; however, other studies are necessary to verify this hypothesis.

Improvement of pig performance is theoretically linked to pig gut health [30]. The diarrheal syndrome is characterized by the loss of electrolytes and water in liquid and semiliquid stools [31]. And the sodium is the main extracellular ion in the body and influences the electrolytic balance of animals [32]. Thus, in our study, there was a higher diarrheal incidence of piglets in the T0 group (Figure 2(b)), and there was a decrease in the serum concentration of Na^+^ of piglets (Table 3) in this group, correspondingly. On the other hand, the oral administration with *Lactobacillus plantarum* CAM6 in sows decreased the diarrheal incidence of their piglets indicating the benign intestinal ecosystem and healthy gut physiology in this group (T1) which could benefit the maintenance of the electrolyte balance in the piglets' bodies [33]. In the meantime, the significant higher pCO_2_ and BHB in the serum of piglets in the T1 group were observed. Although the increase of CO_2_ pressure in piglets may indicate possible metabolic acidosis which could increase *β*-hydroxybutyrate [34], it does not imply an alteration in acid-base balance since gap anion which was within the range biologically expected for the healthy piglets [16]. In this study, all the hematological parameters of lactating piglets were within normal ranges [15, 16], but it is still worth noting that the leukocytes, lymphocytes, and platelets increased in the piglets of the T1 group (*P* ≤ 0.05) compared to T0. A significant increase in leukocytes and lymphocytes in piglets in the T1 group (*P* ≤ 0.05) could indicate that probiotics supplied with *Lactobacillus plantarum* CAM6 in sows might increase the immune response of their piglets. The peptidoglycans and lipopolysaccharides attached to the cell wall of the lactic acid bacteria (LAB) play an important role in the immune system, which increases the number of the precursor cells of the humoral immunity [35]. Liu et al. and Xin et al. reported an increase in endogenous antioxidant enzymes and sIgA when evaluating the effect of yeasts and LAB in piglets [36, 37]. The results in the hematological indicators related to the blood immunological parameters of the present study (Table 4) could be associated with the probiotic microorganisms introduced in the diet of sows which may favor the physiological changes in piglets possibly owing to the homeostasis of the autochthonous microbiota of the gastrointestinal tract and benefiting their healthy and productive performance [38, 39].

## 5. Conclusions

The oral administration of *Lactobacillus plantarum* CAM6 in sows from the last third of gestation until weaning did not affect the behavior of the sow; it decreased the number of death of the piglets before weaning and the diarrheal syndrome and improved the weight gaining performance of their offspring weekly. Furthermore, this probiotic improved the nutritional value of the breast milk consumed by their descendants. Likewise, the group of oral administration of *Lactobacillus plantarum* CAM6 in sows increased Na^+^, D-*β*-hydroxybutyrate, leukocytes, and lymphocytes in their offspring's blood. Oral administration of *Lactobacillus plantarum* CAM6 in breeding sows improved body weight, physiological status, and the health of their offspring.

## Figures and Tables

**Table 1:** Ingredients for sows in pregnancy and lactation and suckling piglets.

Ingredients (%)	Pregnancy	Lactation	Prestarter
Cornmeal	53.40	63.00	36.00
Soymeal	12.60	26.00	20.00
Wheat bran	30.00	4.00	—
Soybean oil	—	3.00	2.00
Sugar			2.00
Gestation nucleus^1^	4.00	—	
Lactation nucleus^2^	—	4.00	
Prestarter nucleus^3^			40.00

^1^Gestation nucleus per kg of product: folic acid 39 mg; pantothenic acid 300 mg; biotin 5 mg; Ca 175500 mg; Co 5 mg; Cu 250 mg; choline 10520 mg; Fe 2150 mg; P 12000 mg; I 25 mg; Mn 1,250 mg; niacin 800 mg; Se 9 mg; Na 48,000 mg; vit. A 250000; vit. B1 60 mg; vit. B12 600 mcg; vit. B2 150 mg; vit. B6 80 mg; vit. C 1250 mg; vit. D3 50000 IU; vit. E 1250 mg; vit. K3 100 mg; and Zn 3125 mg. ^2^Lactation nucleus per kg of product: folic acid 37.5 mg; pantothenic acid 300 mg; BHT 3,750 mg; biotin 5 mg; Ca 205000 mg; Co 6 mg; Cu 250; choline 10000 mg; Fe 2000 mg; P 51000 mg; I 25 mg; Mn 1250 mg; niacin 800 mg; Se 9 mg; Na 44000 mg; vit. A 250000 IU; vit. B1 60 mg; vit. B12 600 mcg; vit. B2 150 mg; vit. B6 80 mg; vit. C 1250 mg; vit. D3 50000 IU; vit. E 1250 mg; vit. K3 100 mg; and Zn 3125 mg. ^3^Pre-starter nucleus: whey powder, milk powder, skim milk powder, choline, extruded soybeans, corn, sugar, fumaric acid, vegetable oil, dicalcium phosphate, threonine, tryptophan, calcite, mineral premix, L-lysine, vitamin premix, sodium chloride, DL-methionine, butyl-hydroxytoluene (BHT); levels per kg of product: vit. A 360,000 IU; vit. D3 7,500 IU; vit. E 450 mg; vit. K3 18 mg; B1 12 mg; B2 29.5 mg; B6 13.5 mg; vit. B12 0.01 mg; niacin 118 mg; pantothenic acid 47.5 mg; folic acid 3.25 mg; biotin 0.75 mg; vit. C 300 mg; choline 1,800 mg; Fe 875 mg; Cu 625 mg; Mn 180 mg; Zn 625 mg; and Co 3.25 mg.

**Table 2:** Effect of oral supplementation of *Lactobacillus plantarum* CAM6 on lactating sows.

Items	*L. plantarum*CAM6		SEM±	*P* value
	T0	T1		
BW 75 days of pregnancy (kg)	229.67	221.00	6.748	0.130
BW at farrowing (kg)	214.97	212.25	5.529	0.201
BW at weaning (kg)	200.00	196.82	3.909	0.690
Weight loss (kg)	14.97	15.43	1.441	0.789
Days of gestation (days)	114.5	115.0	0.360	0.239
Live pigs at birth/litter	12.50	12.00	0.963	0.797
Pigs dead at birth/litter	0.75	1.00	0.126	0.941
Pigs weaned/litter	9.50	9.75	0.381	0.394
Dead pigs before weaning/litter	3.00	2.25	0.226	0.041
Weaning-estrus interval	7.3	6.9	0.417	0.872

BW: body weight; T0: basal diet (BD); T1: BD + 10 mL biological agent containing 10^9^ CFU/mL *L. plantarum* CAM6.

**Table 3:** The derived influence of *Lactobacillus plantarum* CAM6 supplementation in sows' feeding on the BAS and serum BHB of the lactating piglets (28 days of lactation).

Items	*L. plantarum* CAM6		SEM±	*P* value
	T0	T1		
pH	7.34	7.32	0.044	0.819
pCO_2_ (mm/Hg)	44.75	52.50	2.581	0.027
Na (mmol/L)	143.75	147.50	1.325	0.039
K (mmol/L)	4.925	5.350	0.347	0.420
Cl (mmol/L)	106.25	107.00	1.717	0.768
HCO_3_^−^ (mEq/L)	21.98	23.63	1.078	0.321
Anion gap	20.75	22.25	1.216	0.417
D-*β*-Hydroxybutyrate	1.50	2.02	0.154	0.038

T0: basal diet (BD); T1: BD + 10 mL biological agent containing 10^9^ CFU/mL *L. plantarum* CAM6; pCO_2_: CO_2_ pressure; HCO_3_^−^: bicarbonate ion.

**Table 4:** The derived influence of oral administration with *Lactobacillus plantarum* CAM6 in sows towards the hematological parameters of their lactating piglets (28 days of lactation).

Items	*L. plantarum*CAM6		SEM±	*P*-value	Normal parameters^∗^
	T0	T1			
Leukocytes (10^3^/mm^3^)	20.73	24.03	0.804	0.046	5.4-25
Lymphocytes (10^3^/mm^3^)	3.98	5.30	0.420	0.038	3.8-14
Monocytes (10^3^/mm^3^)	1.40	1.78	0.172	0.173	0.001-5.0
Granulocytes (10^3^/mm^3^)	15.35	16.95	3.502	0.758	2.5-23
Eosinophils (10^3^/mm^3^)	0.61	0.66	0.204	0.868	0.0-1.8
Erythrocytes (10^6^/mm^3^)	5.77	5.88	0.473	0.869	4.8-7.3
Hb (g/dL)	8.75	9.23	0.421	0.456	8.8-12.7
Hto (%)	28.78	30.93	1.787	0.428	28-42.7
MCV (*μ*m^3^)	50.00	53.00	1.414	0.184	38-59
MCH (pg)	15.28	15.86	0.620	0.519	11-18
CMCH (g/dL)	30.45	29.98	0.480	0.513	27-32
Platelet (10^3^/mm^3^)	872.52	761.75	28.385	0.041	208-873
MPV (*μ*m^3^)	11.15	10.85	0.792	0.798	7.4-16.5

^∗^Friendship et al. [14]; Cooper et al. [15]. T0: basal diet (BD); T1: BD + 10 mL biological agent containing 10^9^ CFU/mL *L. plantarum* CAM6; Hb: hemoglobin; Hto: hematocrit; MCV: mean corpuscular volume MCH: mean corpuscular hemoglobin; CMCH: concentration of mean corpuscular hemoglobin; MPV: mean platelet volume.

## Data Availability

The data are all included in the manuscript.

## Abbreviations

BAS: Basic acid state
BD: Basal diet
BHB: D-*β*-Hydroxybutyric acid
BHT: Butyl-hydroxytoluene
BSH: Bile salt hydrolase
BW: Body weight
DI: Diarrhea incidence
CMCH: Concentration of mean corpuscular hemoglobin
Hb: Hemoglobin
Hto: Hematocrit
LAB: Lactic acid bacteria
MCH: Mean corpuscular hemoglobin
MCV: Mean corpuscular volume
MPV: Mean platelet volume
pCO_2_: CO_2_ pressure.
